# The Journal

**Published:** 1989-11

**Authors:** 


### The Journal

This may be the last number bearing the old and honourable title *Bristol Medico-Chirurgical Journal*. Readers will know that the Bristol Medico-Chirurgical Society has always aspired to produce a medical journal to serve the whole of the West of England and has now decided that this aim will be best achieved as a joint effort with other West of England medical groups. Delegates from these groups met at Moretonhampstead in July to discuss the project and agreed to recommend it to constituent meetings of their societies. The votes from societies are coming in, so far all have been in favour with a total membership of about 1500. If the remainder also agree to join the association the distribution of the journal will come to about 3000 and will cover the whole of the West of England. The content of this number is happily biased towards Bristol and contains a delightful and very personal Presidential Address from a native of Bristol and a biographical article on Carey Coombs, the pioneer Bristol cardiologist whose memory deserves to be kept bright. Suffice it to say at this moment that we hope that the future of this journal is to be the stem on which a new tree is grafted to flourish in a wider field. If this comes to pass it will be made possible with the sponsorship of the Nuffield Nursing Homes Trust.

